# Cultivating Self-Compassion to Improve Social Workers’ Professional Quality of Life in Primary Healthcare

**DOI:** 10.3390/healthcare13111313

**Published:** 2025-06-01

**Authors:** Polyxeni Mangoulia, Nektarios Tsokas, Evmorfia Koukia, Foteini Malli, Evdokia Missouridou, Chrysoula Dafogianni, Maria Kyranou, Evangelos C. Fradelos

**Affiliations:** 1 Department of Nursing, National and Kapodistrian University of Athens, 115 27 Athens, Greece; ekoukia@nurs.uoa.gr; 2 Mobile Mental Health Unit of Trikala “Syneirmos”, 42 100 Trikala, Greece; nektarios.tsokas@gmail.com; 3 Department of Nursing, University of Thessaly, 41 500 Larissa, Greece; mallifoteini@yahoo.gr (F.M.); evagelosfradelos@hotmail.com (E.C.F.); 4 Department of Nursing, University of West Attica, 12 243 Athens, Greece; emis@uniwa.gr (E.M.); cdafog@uniwa.gr (C.D.); 5 Department of Nursing, Cyprus University of Technology, 3036 Limassol, Cyprus; maria.kyranou@cut.ac.cy

**Keywords:** burnout, compassion fatigue, compassion satisfaction, professional quality of life, secondary traumatic stress, self-compassion, social workers, primary healthcare

## Abstract

Background/Objectives: Although self-compassion practice has been increasingly popular in the literature in recent years, the area of social work knows very little about it. To be self-compassionate is to treat oneself with love and respect. The aim of this study was to investigate the relationship between social workers’ professional quality of life (compassion satisfaction, secondary traumatic stress, and burnout) and self-compassion. Methods: This cross-sectional descriptive study consisted of 177 social workers in primary healthcare (PHC) in Greece. The Self-Compassion Scale (SCS), the Professional Quality of Life Scale (ProQOL-5), and the participants’ personal, demographic, and professional information were all included in an electronic questionnaire that they had to fill out. Results: This study’s findings show that, although 43.2% of participants exhibited high levels of self-compassion, burnout and secondary traumatic stress were at low-to-moderate levels among the participants. Apart from the secondary traumatic stress and humanity dimensions, both the dimensions and the overall self-compassion scale showed a strong relationship with the aspects of professional quality of life. Burnout and secondary traumatic stress were significantly reduced, and compassion fulfilment were increased in environments that were supportive, socially supportive, work-related, hobby-filled, and religious. Conclusions: The integration of self-compassion into a comprehensive and methodical approach to employee wellness has the potential to enhance the physical and mental health of workers in healthcare environments.

## 1. Introduction

Primary healthcare (PHC) serves as the foundation of healthcare systems, ensuring accessible and community-based medical and psychosocial support [1]. Social workers in PHC settings address social determinants of health, advocate for vulnerable populations, and provide emotional and practical support to individuals and families navigating complex health challenges [2]. Their role is pivotal in ensuring equitable access to healthcare services, particularly for marginalized groups experiencing socioeconomic disparities, chronic illnesses, or mental health conditions [3].

Despite their critical contributions, social workers in PHC operate in high-stress environments that expose them to chronic emotional exhaustion, heavy caseloads, bureaucratic challenges, and frequent encounters with client trauma [4]. The cumulative emotional burden of continuously supporting individuals in distress often leads to burnout (BO), secondary traumatic stress (STS), and compassion fatigue (CF) [5,6]. BO is characterized by the emotional exhaustion, depersonalization, and reduced professional efficacy that results from prolonged workplace stress [7]. STS, a core component of compassion fatigue, emerges when professionals internalize the trauma of those they serve, manifesting in intrusive thoughts, avoidance behaviors, and heightened emotional distress [8]. Compassion fatigue, a broader construct, encapsulates both burnout and STS, leading to an erosion of professional well-being [3].

Given the emotional intensity of social work, self-compassion has emerged as a potential protective factor against occupational distress [9]. In primary healthcare settings, social workers face high rates of BO, which is characterized by emotional exhaustion and reduced efficacy; STS, which represent the symptoms arising from indirect trauma exposure, and compassion satisfaction (CS), which is the positive fulfillment derived from helping others. These constructs have been shown to interact dynamically, with higher CS buffering against BO and STS in similar healthcare populations [10,11,12]. Defined as treating oneself with kindness during difficult moments, self-compassion encompasses three key components: self-kindness, common humanity (recognizing that suffering is a shared human experience), and mindfulness (maintaining balanced awareness of negative emotions rather than over-identifying with them) [9,13].

Research suggests that higher levels of self-compassion correlate with reduced burnout, lower emotional exhaustion, and greater psychological resilience in helping professionals [14,15]. Shapiro et al. [16] found that an eight-week mindfulness-based intervention significantly reduced stress symptoms and improved well-being among social workers. Similarly, Duarte et al. [17] demonstrated that self-compassion enhances emotional regulation, mitigating the adverse effects of compassion fatigue. Additionally, findings indicate that self-compassionate practitioners experience higher levels of job satisfaction and are more likely to sustain long-term engagement in their profession [18].

Despite these promising insights, self-compassion remains underexplored among PHC social workers. While the role of self-compassion in preventing burnout has been widely examined among physicians and nurses [10,11], there is limited empirical evidence on its impact among PHC social workers, despite their equally demanding roles.

However, studies on physicians and other healthcare professionals have highlighted its benefits in reducing emotional exhaustion and enhancing professional well-being [10,11]. Research specifically targeting social workers, particularly in the primary healthcare setting, is limited [12]. Moreover, evidence suggests that many social workers struggle to implement self-care practices due to systemic barriers, heavy workloads, and professional expectations to prioritize client needs over personal well-being [3].

Stamm [6] offers a comprehensive model for understanding occupational well-being in helping professions through the Professional Quality of Life (ProQOL) framework, which consists of three dimensions:

- CS: The emotional fulfillment derived from helping others, and it is associated with increased motivation and professional engagement.
- BO: Emotional exhaustion and cynicism resulting from prolonged workplace stress.
- STS: Trauma-related symptoms emerging from repeated exposure to clients’ suffering, leading to emotional detachment or distress.

A growing body of research acknowledges the importance of self-care, emotional resilience, and workplace interventions in mitigating compassion fatigue among social workers. However, several key gaps persist in the literature. Few studies have explicitly examined self-compassion as a protective factor, despite its established benefits in other healthcare professions [9,19], for PHC social workers. Limited research has explored the relationship between self-compassion and professional quality of life (CS, BO, and STS) in social workers, particularly within high-stress environments like PHC [3]. Despite recommendations for self-care and resilience-building strategies, social workers report difficulty integrating self-compassion into their daily practice, highlighting a need for further investigation into systemic and individual barriers [2].

The first reference to the terms professional quality of life, secondary traumatic stress, compassion fatigue, and compassion satisfaction in the Greek literature, as well as the first study conducted on a Greek population (nurses), was in the doctoral dissertation of Mangoulia [20]. However, no studies have systematically analyzed these terms and the role of self-compassion in Greek PHC social workers.

However, despite its proven benefits in nursing and medicine, self-compassion’s role in modulating compassion satisfaction, burnout, and STS among social workers in Greek primary healthcare (PHC) remains underexplored.

More specifically, in Greece, PHC has undergone major reforms since 2017 to expand accessibility and integrate multidisciplinary teams into community settings [21]. Despite these advances in PHC organization, few studies have specifically examined self-compassion among social workers in this evolving context. At the same time, comparable European health systems have reported similar challenges in workforce well-being and service delivery [22]. Therefore, our findings have implications not only for ongoing Greek PHC reform efforts, but also for analogous primary care models across Europe.

This study aims to examine the association between self-compassion and professional quality of life (compassion satisfaction, burnout, and secondary traumatic stress) among social workers in Greek primary healthcare (PHC) settings. Specifically, it seeks to examine how self-compassion correlates with compassion satisfaction, burnout, and secondary traumatic stress; to analyze whether individual, demographic, and professional characteristics influence these relationships; and to explore the potential of self-compassion as a self-care strategy for social workers, emphasizing its role in fostering professional sustainability and reducing emotional exhaustion.

By addressing this understudied area, the findings of this study may contribute to the development of targeted interventions and policy recommendations aimed at enhancing social workers’ resilience, well-being, and professional sustainability in PHC settings.

## 2. Materials and Methods

### 2.1. Data Collection

Research data were gathered online through a structured, closed-ended questionnaire administered via Google Forms after obtaining approval from each regional health authority in Greece. Online data collection offers an efficient and accessible means of reaching a target population while minimizing geographical and logistical constraints. Data collection was conducted between October and December 2023. Completing the questionnaire required approximately 15 min.

A convenience sampling strategy was implemented with a method recognized for its practicality in acquiring a research sample within a limited timeframe [23]. Participants were recruited through two main channels:

1. Emails sent to regional health authorities that granted permission; some authorities automatically forwarded the survey link to their employed PHC social workers, while others provided access upon request by individual social workers.
2. The Association of Social Workers of Greece, which distributed the questionnaire to its members, explicitly stating that it was intended only for social workers employed in primary healthcare settings. A screening question at the beginning confirmed participants’ current role and place of employment in PHC, automatically excluding any ineligible responses.

### 2.2. Participants

The target population comprised professional social workers employed in PHC structures across Greece. A total of 177 social workers participated (mean age = 38.7 ± 8.9 years; 88.7% female; mean professional experience = 12.3 ± 7.1 years).

No demographic restrictions were applied during the sampling process, allowing for the inclusion of participants across various age groups, genders, and types of PHC structures. This inclusive approach aimed to enhance the generalizability, reliability, and validity of the study’s findings.

Prior to participation, all respondents were provided with detailed information about the study’s objectives and procedures, ensuring voluntary participation and confidentiality of their responses.

### 2.3. Research Instrument

A structured questionnaire consisting of three sections was utilized for data collection.

Section 1: Demographic, Individual, and Professional Characteristics

This section comprised 23 items assessing key demographic and professional variables, including gender, age, marital status, number of children, education level, and years of professional experience. Additionally, it explored personal and social aspects, such as hobbies, relationships with family and friends, experience of traumatic events, the presence of chronic illnesses in the family, workplace environment, level of support, spiritual care, and belief in God.

Section 2: Self-Compassion Assessment

Self-compassion was measured using the Self-Compassion Scale (SCS), which was developed by Neff in 2003 [9], translated into Greek by Mantzios et al. [24], and has established validity for the Greek population. The scale consists of 26 self-reported items rated on a five-point Likert scale (1 = almost never to 5 = almost always), with higher scores indicating greater self-compassion.

The SCS assesses both positive (Self-Kindness, Common Humanity, and Mindfulness) and negative (Self-Judgment, Isolation, and Over-Identification) dimensions of self-compassion. Higher self-compassion levels are generally associated with greater psychological well-being, as individuals who practice self-kindness, universality, and mindfulness tend to experience improved mental health. Importantly, self-compassion differs from self-esteem and narcissism. While high self-esteem relates to self-worth and self-respect, narcissism is characterized by excessive egocentrism and a lack of emotional connection with others [9].

Section 3: Professional Quality of Life Assessment

The Professional Quality of Life Scale (ProQOL-5) was used to evaluate professional well-being [6]. The fifth revised edition of the ProQOL-5, which has been translated and validated in Greek [25,26], was employed. In our sample, the Cronbach’s α for the Self-Compassion Scale (SCS) was 0.90 compared to the 0.80 in the original Greek validation [22]. For the ProQOL-5 subscales, we obtained an α = 0.90 for compassion satisfaction, 0.75 for burnout, and 0.74 for secondary traumatic stress, closely matching the published Greek values [25,26] and confirming excellent internal consistency in our data. These α coefficients exceed the commonly accepted threshold of 0.70, justifying our choice of SCS and ProQOL-5 for this study.

The scale comprised 30 self-reported items rated on a five-point Likert scale (1 = almost never to 5 = almost always), and it measured three distinct dimensions: compassion satisfaction (positive aspects of caregiving), burnout (emotional exhaustion and reduced personal accomplishment), and secondary traumatic stress (stress related to indirect exposure to trauma). Burnout and secondary traumatic stress were conceptualized as compassion fatigue. According to the official ProQOL manual [6], interpretation of scores is guided by normative percentiles. Specifically, scores below the 25th percentile are categorized as low, scores between the 25th and 75th percentiles as moderate, and scores above the 75th percentile as high. These ranges are not diagnostic but serve as indicators of relative risk or resilience in professional quality of life. This classification was applied in the analysis of participants’ results in the present study.

### 2.4. Ethical Considerations

This study received approval from the Ethics and Deontology Committee of the University of Thessaly (Approval Protocol Number 491/20-09-2023). All participants, ensuring that their responses remained strictly confidential and anonymous, thus safeguarding their personal data, provided informed consent.

Formal approval was also obtained from the respective scientific councils of the regional health authorities overseeing primary healthcare structures before this study was conducted. The approval details are as follows: 1st Health Region: Approval Protocol Number 44471/13-11-2023, 5th Health Region: Approval Protocol Number 88260/28-11-2023, and 6th Health Region: Approval Protocol Number 63516/18-12-2023.

Throughout the study, the anonymity and confidentiality of all personal information were strictly maintained, and no financial burden was placed on participating in primary healthcare structures. Additionally, the research process was facilitated through collaboration with the Association of Social Workers of Greece, whose Board of Directors approved the procedure (Approval Protocol Number 162/23-01-2024).

All measurement scales used in the questionnaire were publicly available in their translated versions online, and permission was sought from the original authors where applicable. The research adhered fully to the ethical guidelines set forth in the Declaration of Helsinki and Law 4624/2019 on the “Personal Data Protection Authority”.

### 2.5. Statistical Analysis

The Kolmogorov–Smirnov test was employed to examine the distribution of the quantitative variables in terms of normality. For variables that followed a normal distribution, mean values and standard deviations (SD) were used for their description. For the description of qualitative variables, absolute frequencies (N) and relative frequencies (%) were reported.

To compare the quantitative variables between two groups, Student’s *t*-test was applied. For comparisons of the quantitative variables among more than two groups, the parametric analysis of variance (ANOVA) was used. To assess the relationship between two quantitative variables, the Pearson correlation coefficient was calculated.

Linear regression analysis with a stepwise procedure was utilized to identify the independent factors associated with the Professional Quality of Life scales and the Self-Compassion scale. The resulting regression coefficients (β) and their corresponding standard errors (SE) were reported. All tests were two-tailed, with a significance level set at *p* < 0.05. The data were analyzed using SPSS 26.0 statistical software (IBM Corp., Armonk, NY, USA).

## 3. Results

### 3.1. Demographic Characteristics of the Participants

The sample consists of 177 primary healthcare social workers, 41.8% of whom belonged to the age group of 30–39 years. Most participants (88.7%) were women. Additionally, 41.8% were TE social workers (level of study: Technological Educational Institute), 50.3% were married, and 49.7% had no children. A significant percentage (44.1%) resided in a large provincial city, while 52% had an annual household income not exceeding EUR 15,000. Table 1 presents the demographic characteristics of the participants, and Table 2 shows their personal and professional characteristics.

The study findings indicate that 37.9% of participants had between one and five years of experience as social workers. Additionally, 50.3% were employed in either private or public sector organizations, excluding health centers, health units, or regional medical clinics. For 60.5% of respondents, their work involved both children and adults. Furthermore, 75.8% described the workplace environment as good to very good. Lastly, 67.8% provided spiritual care as part of their professional duties.

Regarding health conditions, 10.2% reported having one chronic illness, while 4% had two chronic conditions. Additionally, 16.9% lived with someone suffering from a chronic illness, and 61% had experienced the death of a loved one in recent years. Moreover, 36.2% had encountered a traumatic event in their lifetime that threatened their life or physical integrity.

In terms of social relationships, 80.7% reported having good-to-very-good relationships with their family, while 91% reported similarly positive relationships with their friends. Additionally, 85.3% stated that they received good-to-very-good support from those around them. Furthermore, 65% derived strength from God or another higher power to some extent or entirely, while 76.3% engaged in a hobby that brought them joy. Finally, 32.8% had sought help from a mental health professional in the past, and 18.6% were currently receiving such support.

### 3.2. Professional Quality of Life (ProQOL) Results

Table 3 presents the results for the Professional Quality of Life scale, which consists of two subscales: one positive and one negative. The positive subscale is “Compassion Satisfaction”, while the negative subscale is “Compassion Fatigue”, which is further divided into “Burnout” and “Secondary Traumatic Stress”.

The Kolmogorov–Smirnov test confirmed the normal distribution of the quantitative variables “Compassion Satisfaction”, “Burnout”, and “Secondary Traumatic Stress” (*p* > 0.005 for all three dimensions). Scores in all dimensions ranged from 10 to 50 points. Specifically, a score of 22 or lower in any dimension indicate low levels of satisfaction, burnout, and secondary traumatic stress, while scores between 23 and 41 suggest moderate levels. Finally, scores above 42 indicate high levels of these constructs.

The Cronbach’s α reliability coefficient was greater than 0.7 across all dimensions, indicating acceptable reliability. The majority of participants reported moderate compassion satisfaction (58.2%) and low levels of burnout (53.1%) and secondary traumatic stress (52%). Notably, no participants exhibited high levels of burnout or secondary traumatic stress. The corresponding percentages for moderate burnout and moderate secondary traumatic stress were 46.9% and 48%, respectively.

### 3.3. Self-Compassion Scale (SCS) Results

Table 4 presents the results for the dimensions and the overall self-compassion scale. Both the individual dimensions and the total scale ranged from 1 to 5 points. The Kolmogorov–Smirnov test was used to assess the distributions of the dimensions and the overall self-compassion scale. The normality hypothesis was rejected for the dimensions (*p* < 0.005 for all), whereas the overall scale followed a normal distribution (*p* = 0.200). The necessary reverse coding of specific items was applied to ensure that each dimension had a positive interpretation in the scoring process.

The “Self-kindness” dimension ranged from 1.4 to 5 points with a mean score of 3.6 (SD = 0.7), indicating moderate-to-high levels of self-kindness. The “Common Humanity” dimension ranged from 1.3 to 5 points with a mean score of 3.3 (SD = 0.7), suggesting a moderate tendency to perceive oneself as part of a common humanity. The “Mindfulness” dimension ranged from 1.5 to 5 points with a mean score of 3.7 (SD = 0.7), reflecting moderate-to-high levels of mindfulness.

The dimensions “Self-judgment”, “Overidentification”, and “Isolation” ranged from 1.0 to 5 points with mean scores of 3.3 (SD = 0.8), 3.1 (SD = 0.9), and 3.4 (SD = 0.9), respectively, indicating moderate levels of self-criticism, overidentification, and isolation. The overall self-compassion scale ranged from 1.8 to 4.6 points with a mean score of 3.4 (SD = 0.6), suggesting moderate levels of self-compassion. The Cronbach’s α reliability coefficient was greater than 0.7 for all dimensions and the overall scale, indicating acceptable reliability.

Among the participants, 53.7% exhibited high levels of self-kindness, 33.9% reported high levels of common humanity, and 54.8% demonstrated high mindfulness. Additionally, 37.9% did not engage in high levels of self-criticism, 40.1% experienced low levels of isolation, and 29.9% avoided excessive overidentification. Overall, 43.2% of participants displayed high levels of self-compassion.

### 3.4. Correlation Between the Dimensions of the Professional Quality of Life with Self-Compassion Scale

Table 5 presents the correlation coefficients for the dimensions of the Professional Quality of Life scale with the dimensions and overall score of the Self-Compassion Scale, as well as the personal and professional characteristics.

Both the dimensions and the total score of self-compassion were significantly associated with the dimensions of professional quality of life, except for the humanity dimension and the secondary traumatic stress dimension. Specifically, individuals who exhibited greater self-kindness experienced increased humanity and mindfulness, along with reduced self-criticism, feelings of isolation, and overwhelming negative emotions. These changes were associated with higher compassion satisfaction, lower burnout, and less severe secondary traumatic stress.

Moreover, participants with stronger relationships within their social environment experienced higher compassion satisfaction and lower burnout. The same pattern was observed for work climate: a more positive work environment was associated with increased compassion satisfaction and reduced burnout. Additionally, individuals who reported faith in God or a higher power had lower burnout scores. Finally, greater social support from one’s surroundings was significantly related to increased compassion satisfaction and a notable reduction in both burnout and secondary traumatic stress.

### 3.5. Association Between the Professional Quality of Life and Personal/Professional Characteristics

According to the results of the Student’s *t*-test and ANOVA, the dimensions of professional quality of life did not differ based on the participants’ personal and professional characteristics (gender, age, education level, marital status, place of residence, annual family income, years of experience as a social worker, total years of work experience, workplace in primary healthcare, and the population they work with—adults or children and adolescents).

Table 6 presents the participants’ scores on the dimensions of ProQOL based on their management of chronic illnesses, cohabitation with individuals with chronic conditions, and their personal experiences and beliefs. Those living with someone with a chronic illness had higher levels of burnout compared to those who did not cohabit. Furthermore, those who had experienced the death of a loved one in recent years reported lower burnout compared to those who had not. Lastly, having a hobby that brings joy was associated with reduced secondary traumatic stress. The presence of a chronic illness or traumatic event threatening their life or integrity, as well as psychological support from a mental health professional, did not show any statistically significant difference for any of the three dimensions of ProQOL.

### 3.6. Multiple Linear Regression Analysis Results for Professional Quality of Life

To identify the factors independently associated with the dimensions of professional quality of life, multiple linear regressions were conducted, with the scores on the three dimensions set as dependent variables and the demographic, work-related, and personal characteristics of the participants, as well as the self-compassion scale, set as independent variables. The results of the analyses using the stepwise method are presented in Table 7.

The self-compassion scale and work climate were found to be independently associated with the dimension of compassion satisfaction. Specifically, greater self-compassion was associated with higher satisfaction with compassion from others. A better work climate was also associated with higher compassion satisfaction. The model with these independent predictor variables explained 51% of the variability in the compassion satisfaction dimension.

The self-compassion scale, work climate, social support, and cohabitation with someone suffering from a chronic illness were found to be independently associated with the dimension of burnout. Specifically, greater self-compassion was associated with lower burnout. A better work climate was also linked to lower burnout. Greater social support was associated with reduced burnout. Those living with someone who suffers from a chronic illness had higher burnout compared to those who did not cohabit. The model with these independent predictor variables explained 67% of the variability in the burnout dimension.

The self-compassion scale, social support, and engagement in hobbies were found to be independently associated with the dimension of secondary traumatic stress. Specifically, greater self-compassion was associated with reduced secondary traumatic stress. Greater social support was also associated with reduced secondary traumatic stress. Those who had a hobby that brought them joy reported less traumatic stress compared to those who did not engage in hobbies. The model with these independent predictor variables explained 52% of the variability in the secondary traumatic stress dimension.

## 4. Discussion

### 4.1. Discussion of the Results

This study established self-compassion as a crucial protective factor against occupational stress and burnout among social workers. Consistent with prior research [27,28], our findings suggest cultural factors in Greece, such as strong familial and community support systems, may foster resilience among social workers [16]. Social factors, including the collaborative nature of multidisciplinary teams in PHC settings, appear to buffer against occupational stress [17,18]. Systemic factors, such as ongoing healthcare reforms and resource reallocation since 2017, may have both challenged and incentivized adaptive coping strategies among practitioners. Practitioners who cultivate self-compassion experience lower acute stress, reduced emotional exhaustion, and a decreased likelihood of burnout. Notably, the observed negative correlation between self-compassion and burnout (r = −0.45, *p* < 0.001) underscores how resilience factors translate into tangible reductions in professional distress. Similarly, the positive association with compassion satisfaction (r = 0.52, *p* < 0.001) highlights the practical benefits of self-compassion interventions in PHC settings. These results reinforce the notion that self-compassion is not merely a theoretical construct but a practical psychological resource for professionals working in emotionally demanding environments.

Our findings align with previous studies supporting mindfulness-based interventions for social workers [29]. Both our study and theirs emphasize the effectiveness of integrating mindfulness and self-compassion training into professional development as a promising strategy for mitigating job-related stress and burnout. However, differences in methodologies, participant characteristics, and assessment tools across studies suggest that the impact of mindfulness-based self-compassion programs may vary depending on contextual factors, warranting further investigation in diverse populations.

Beyond social work, several studies in healthcare settings reinforce our conclusions. Multiple quantitative studies have demonstrated the protective effects of self-compassion under varying conditions and among different professional groups. For instance, Durkin et al. [30] found that higher levels of self-compassion correlated with lower burnout rates among UK community nurses. Similarly, Beaumont et al. [15] reported that student midwives with greater self-compassion exhibited improved mental well-being, reduced burnout, and lower compassion fatigue. These findings parallel our own, suggesting that self-compassion’s benefits extend beyond social work to other healthcare professions. Furthermore, Kotera et al. [31] identified self-compassion as a key predictor of better mental health among social work students, highlighting the potential for resilience-building through self-compassion training early in professional development. Moreover, Miller et al. [12] demonstrated that self-compassion significantly predicts better self-care practices among social work clinicians, further highlighting its relevance as a practical tool for occupational well-being.

Recent literature has expanded on the role of self-compassion in professional well-being. Neff and Knox [32] developed an adapted version of the Mindful Self-Compassion program for healthcare providers, demonstrating that this training significantly reduced burnout and improved resilience. Similarly, Lyon and Wright [28] found that integrating mindfulness and self-compassion into psychology education not only mitigated burnout, but also enhanced overall psychological well-being. Structured self-compassion interventions have also proven effective in reducing distress and strengthening coping mechanisms among school counselors [33]. Collectively, these studies contribute to the growing body of evidence supporting self-compassion training as a practical intervention for occupational well-being across helping professions.

While self-compassion appears to be universally beneficial, its effectiveness may vary depending on specific workplace demands. Mangoulia found that secondary traumatic stress and burnout levels were significantly higher among ICU and psychiatric nurses compared to social workers in rural clinics [20]. This discrepancy is likely due to differences in trauma exposure and job demands, reinforcing the idea that the workplace environment influences stress levels. While self-compassion is a valuable tool for managing workplace stress, its protective effects may be moderated by the intensity and nature of job-related challenges.

### 4.2. Limitations of the Study

Several limitations should be acknowledged when interpreting the findings of this study. One of the primary limitations is the restricted timeframe in which this study was conducted, potentially limiting the ability to collect data from a broader range of professionals and organizational contexts. Time constraints may have also affected participant availability and willingness to engage in the study.

Another key limitation is the geographical coverage of the study. Not all regional units of the country were able to participate, which may affect the generalizability of the results. Variations in organizational structures, workplace culture, and available support systems across different regions could influence the extent to which self-compassion serves as a protective factor against burnout and occupational stress. Future studies should aim for broader geographical representation to enhance the external validity of findings.

A further methodological limitation concerns the self-reported nature of the data. The reliance on self-assessment questionnaires introduces the possibility of response bias, including social desirability effects. Participants may have over- or underestimated their levels of self-compassion, stress, or burnout based on personal perceptions or external expectations. Future research could incorporate objective stress indicators, such as physiological measures (e.g., cortisol levels) or third-party evaluations, to complement self-reported data and provide a more comprehensive assessment.

Additionally, the sampling strategy relied on convenience sampling, which, while practical, may limit the representativeness of the findings. The method increases the risk of selection bias as individuals who voluntarily participate in the study may have different levels of self-compassion or resilience compared to those who opt out. A more randomized or stratified approach in future research would help mitigate this issue and provide a more balanced representation of the target population.

Another consideration is the potential selection bias in participant engagement. Those experiencing severe stress or burnout may have been less likely to take part in this study due to time constraints or emotional exhaustion. As a result, the sample may underrepresent individuals most affected by occupational stress, leading to a possible underestimation of the true impact of self-compassion in highly demanding work environments. Addressing this in future studies could involve targeted outreach efforts to ensure that individuals across all stress and burnout levels are adequately represented.

Despite these limitations, the findings contribute valuable insights into the role of self-compassion in mitigating occupational stress and burnout among social workers. Future research could expand on this work using larger, more diverse samples, employing longitudinal designs to assess causality, and integrating multi-method approaches to strengthen the validity of the results.

### 4.3. Implications for Clinical Practice

Our findings support the integration of self-compassion training into social work education and ongoing professional development. This aligns with the broader literature advocating for proactive self-care strategies among helping professionals [34] and the role of work–life balance in mitigating burnout [35]. Training programs could incorporate evidence-based interventions, such as Mindful Self-Compassion workshops and resilience-building exercises, to enhance practitioners’ well-being. Organizations employing social workers may also consider offering regular self-compassion and mindfulness sessions, utilizing digital platforms to increase accessibility and engagement.

The importance of self-care and resilience in social work has been further underscored by research conducted during the COVID-19 pandemic. Studies have demonstrated that lower resilience and higher burnout levels are associated with reduced quality of care, emphasizing the necessity of workforce well-being to maintain high standards of client service. Sessions et al. (2025) found that experienced social workers tend to exhibit relatively high levels of self-compassion, which correlates with lower psychological distress [34,36]. Interestingly, their review also indicated that social work students generally report lower self-compassion levels than seasoned practitioners, suggesting an opportunity for academic programs to incorporate self-compassion training to better prepare students for the challenges of professional practice.

This study adds to the growing evidence that self-compassion is a critical resource for social workers managing the psychological demands of their profession. Developing self-compassion not only enhances psychological resilience and job satisfaction, but also has the potential to improve retention in the field and the quality of care provided to clients. As organizations and training programs seek to support the well-being of social workers, self-compassion training should be considered not just as an individual self-care practice, but also as a broader strategy for sustaining a healthy and effective workforce in social services. Future research should continue to explore and refine self-compassion interventions, assess their long-term outcomes, and adapt them to the diverse needs of helping professionals in different work environments.

## 5. Conclusions

This study found that the Greek PHC social workers interviewed reported moderate-to-high compassion satisfaction (mean 39.4 ± 6.4), moderate burnout (mean 22.4 ± 5.4), and moderate secondary traumatic stress (mean 22.9 ± 6.2). In particular, 43.2% of participants scored in the moderate–high range for self-compassion, which was significantly associated with higher compassion satisfaction and lower burnout and STS (*p* < 0.001 for all). These results indicate that the overall professional quality of life is acceptable but that targeted interventions are needed to further reduce moderate levels of burnout and STS. We, therefore, recommend implementing structured self-compassion training and enhancing organizational support in PHC settings to improve specific dimensions of professional well-being. Future studies employing longitudinal designs and intervention-based approaches are warranted to evaluate the long-term effectiveness of these measures in the Greek healthcare context.

## Figures and Tables

**Table 1:** Demographic characteristics of the participants (*n* = 177).

		N	%
Sex	Man	20	11.3
	Woman	157	88.7
Age	20–29	38	21.5
	30–39	74	41.8
	40–49	41	23.2
	50 and over	24	13.6
Level of study	TEI	74	41.8
	University	35	19.8
	Postgraduate	65	36.7
	Doctorate	3	1.7
Marital status	Single	62	35.0
	Married	89	50.3
	I live with a partner.	17	9.6
	Divorced–Widowed	9	5.1
Number of children	0	88	49.7
	1	34	19.2
	2	42	23.7
	3	13	7.3
Place of residence	Urban Center (Athens–Thessaloniki)	56	31.6
	Large provincial town	78	44.1
	Town	17	9.6
	Village	10	5.6
	Island	16	9.0
Annual family income	Up to EUR 15.000	92	52.0
	EUR 15.001–EUR 25.000	50	28.2
	EUR 25.001–EUR 35.000	25	14.1
	EUR 35.001–EUR 50.000	8	4.5
	EUR 50.000 and above	2	1.1

**Table 2:** Personal and professional characteristics of the participants (*n* = 177).

		N	%
Years of experience as a social worker	1–5	67	37.9
	6–10	52	29.4
	11–15	13	7.3
	16–20	19	10.7
	21 and over	26	14.7
Years of total work experience	1–10	78	44.1
	11–20	63	35.6
	21–30	26	14.7
	31 and over	10	5.6
Workplace in primary healthcare (PHC)	Health Center	37	20.9
	Health Unit (TOMY, etc.)	50	28.2
	Regional Clinic	1	0.6
	Other private or public body	89	50.3
Your work as a social worker concerns?	Adults	61	34.5
	Children & Adolescents	9	5.1
	Both	107	60.5
How would you characterize the work climate?	Very bad	7	4.0
	Bad	13	7.3
	Neutral	23	13.0
	Good	87	49.2
	Very good	47	26.6
Do you have a chronic illness yourself?	No, no disease.	152	85.9
	Yes, a disease	18	10.2
	Yes, two diseases	7	4.0
Do you live with someone who has a chronic illness?	No	147	83.1
	Yes	30	16.9
I have experienced the death of a loved one in recent years.	No	69	39.0
	Yes	108	61.0
I have experienced a traumatic event in my life where my life or integrity was threatened.	No	113	63.8
	Yes	64	36.2
What is your relationship with your family environment?	Very bad	9	5.1
	Bad	9	5.1
	Neutral	16	9.0
	Good	53	29.9
	Very good	90	50.8
What is your relationship with your friends?	Very bad	11	6.2
	Bad	1	0.6
	Neutral	4	2.3
	Good	49	27.7
	Very good	112	63.3
Do you have a hobby that gives you joy?	No	42	23.7
	Yes	135	76.3
I draw strength in my life from my belief in God or some other higher power.	Not true at all	32	18.1
	Valid to a very small extent	30	16.9
	Valid to some extent	77	43.5
	Absolutely valid.	38	21.5
Have you previously received or are you currently receiving psychological support from a mental health specialist?	Yes, in the past	58	32.8
	Yes, at this time	33	18.6
	No, I have not received it.	86	48.6
How would you characterize the social support you receive from those around you?	Very bad	2	1.1
	Bad	4	2.3
	Neutral	20	11.3
	Good	88	49.7
	Very good	63	35.6

**Table 3:** Professional quality of life results (ProQOL).

	Compassion Satisfaction	Burnout	Secondary Traumatic Stress
Mean (SD)	39.4 (6.4)	22.4 (5.4)	22.9 (6.2)
Cronbach’s α	0.90	0.75	0. 74
Min-Max	17.0–50.0	11.0–37.0	10.0–40.0
	N (%)		
Low	3 (1.7)	94 (53.1)	92 (52)
Moderate	103 (58.2)	83 (46.9)	85 (48)
High	71 (40.1)	0	0

**Table 4:** Self-compassion scale (SCS) results.

	Self-Kindness	Self-Judgment *	Common Humanity	Isolation *	Mindfulness	Overidentification *	Total Self-Compassion Scale
Mean (SD)	3.6 (0.7)	3.3 (0.8)	3.3 (0.7)	3.4 (0.9)	3.7 (0.7)	3.1 (0.9)	3.4 (0.6)
Min-Max	1.4–5.0	1.0–5.0	1.3–5.0	1.0–5.0	1.5–5.0	1.0–5.0	1.8–4.6
Median (intermediate range)	3.6 (3.2–4)	3.2 (2.8–3.8)	3.3 (2.8–3.8)	3.5 (3–4)	3.8 (3.3–4.3)	3 (2.5–3.8)	-
Cronbach’s α	0.82	0.82	0.71	0.74	0.71	0.76	0.80
	N (%)						
Low	14 (7.9)	26 (14.7)	22 (12.4)	19 (10.7)	4 (2.3)	39 (22)	9 (5.1)
Moderate	68 (38.4)	84 (47.5)	95 (53.7)	87 (49.2)	76 (42.9)	85 (48)	91 (51.7)
High	95 (53.7)	67 (37.9)	60 (33.9)	71 (40.1)	97 (54.8)	53 (29.9)	76 (43.2)

* The values have been reversed to have a positive meaning.

**Table 5:** The Pearson correlations coefficients of ProQOL with the SCS and the personal and professional characteristics.

		CS	ΒO	STS
Self-kindness	r	0.51	−0.51	−0.27
	*p*	<0.001	<0.001	<0.001
Common humanity	r	0.22	−0.23	0.13
	*p*	0.004	0.002	0.080
Mindfulness	r	0.42	−0.40	−0.17
	*p*	<0.001	<0.001	0.026
Self-criticism	r	0.29	−0.48	−0.45
	*p*	<0.001	<0.001	<0.001
Isolation	r	0.42	−0.54	−0.45
	*p*	<0.001	<0.001	<0.001
Hyperidentification	r	0.26	−0.43	−0.49
	*p*	<0.001	<0.001	<0.001
Self-compassion scale	r	0.50	−0.62	−0.43
	*p*	<0.001	<0.001	<0.001
What is your relationship with your friends?	r	0.18	−0.30	−0.12
	*p*	0.016	<0.001	0.123
How would you characterize the work climate?	r	0.23	−0.24	−0.07
	*p*	0.003	0.001	0.349
I draw strength in my life from my belief in God or some other higher power.	r	0.10	−0.18	0.11
	*p*	0.193	0.016	0.162
How would you characterize the social support you receive from those around you?	r	0.34	−0.39	−0.34
	*p*	<0.001	<0.001	<0.001

**Table 6:** The associations between ProQOL and personal/professional characteristics.

		CS		BO		STS	
		Mean (SD)	*p*	Mean (SD)	*p*	Mean (SD)	*p*
Do you live with someone who has a chronic illness?	No	39.5 (6.5)	0.585+	21.9 (5.3)	0.005+	22.6 (6.2)	0.155+
	Yes	38.8 (6.1)		24.9 (5.5)		24.4 (6.4)	
I have experienced the death of a loved one in recent years.	No	38.4 (6.8)	0.090+	23.5 (5.5)	0.026+	22.5 (6.9)	0.528+
	Yes	40 (6.1)		21.6 (5.3)		23.1 (5.8)	
Do you have a hobby that gives you joy?	No	37.8 (6.3)	0.072+	22.8 (5.7)	0.545+	25.2 (6.5)	0.006+
	Yes	39.9 (6.4)		22.2 (5.3)		22.2 (6)	

+ Students *t*-test

**Table 7:** Multiple linear regression results for the Professional Quality of Life scale.

	b+	95% CI (b)	SE++	*p*
Dependent variable: compassion satisfaction (R^2^ = 051, F = 30.5, *p* < 0.001)				
Constant	16.414	(10.514, 22.313)	2.989	<0.001
Self-compassion scale	5.331	(3.832, 6.830)	0.759	<0.001
How would you characterize the work climate?	1.251	(0.440, 2.063)	0.411	0.003
Dependent variable: burnout (R^2^ = 0.67, F = 34.45, *p* < 0.001)				
Constant	47.373	(42.694, 52.052)	2.370	<0.001
Self-compassion scale	−5.226	(−6.412, −4.040)	0.601	0.001
How would you characterize the work climate?	−0.847	(−1.462, −0.231)	0.312	0.007
How would you characterize the social support you receive from those around you?	−1.022	(−1.858, −0.186)	0.424	0.017
Do you live with someone who has a chronic illness? (Yes vs. No)	1.748	(0.121, 3.375)	0.824	0.035
Dependent variable: secondary traumatic stress (R^2^ = 052, F = 20.90, *p* < 0.001)				
Constant	45.471	(39.717, 51.226)	2.915	<0.001
Self-compassion scale	−3.969	(−5.523, −2.414)	0.788	<0.001
How would you characterize the social support you receive from those around you?	−1.702	(−2.773, −0.630)	0.543	0.002
Do you have a hobby that gives you joy? (Yes vs. No)	−2.620	(−4.499, −0.741)	0.952	0.007

**+** Regression coefficient ++ standard error.

## Data Availability

Data supporting this study are available from the corresponding authors upon reasonable request.

## Abbreviations

The following abbreviations are used in this manuscript:

ProQOL	Professional Quality of Life
CF	Compassion Fatigue
BO	Burnout
STS	Secondary Traumatic Stress
SCS	Self-Compassion Scale
PHC	Primary Healthcare
